# Meta-analysis of fish consumption and risk of pancreatic cancer in 13 prospective studies with 1.8 million participants

**DOI:** 10.1371/journal.pone.0222139

**Published:** 2019-09-06

**Authors:** Wei Jiang, Min Wang, Hai-Zhong Jiang, Guo-Chong Chen, Yong-Fei Hua

**Affiliations:** 1 Department of Hepatobiliary and Pancreatic Surgery, Ningbo Medical Center Lihuili Eastern Hospital, Ningbo, China; 2 Department of Gastroenterology, Ningbo First Hospital, Ningbo, China; 3 Department of Epidemiology and Population Health, Albert Einstein College of Medicine, Bronx, New York, United States of America; Dartmouth College Geisel School of Medicine, UNITED STATES

## Abstract

A previous meta-analysis suggested no association between fish consumption and risk of pancreatic cancer. As several prospective studies with a large number of pancreatic cancer cases have emerged after that meta-analysis, we updated the evidence and examined the relationship in greater depth. We performed a literature search on PubMed and EMBASE databases through March 30, 2019 to identify potentially eligible studies. We used a random-effects model to compute summary relative risk (RR) with corresponding 95% confidence interval (CI). A total of 13 prospective studies comprising 4994 pancreatic cancer cases and 1,794,601 participants were included in the final analyses. Results of the meta-analysis showed that fish consumption was not significantly associated with risk of pancreatic cancer (RR _50-g/day_ = 1.03, 95% CI: 0.95–1.12), which was confirmed when stratifying the analysis by various methodological and population characteristics. There was a suggestion of difference by adjustment for family history of pancreatic cancer (*P*_difference_ = 0.05), with fish consumption being associated with higher risk of pancreatic cancer in studies without adjustment for participants’ family history (RR_50-g/day_ = 1.09, 95% CI: 1.02–1.18), and a non-significant inverse association among studies with the adjustment (RR_50-g/day_ = 0.93, 95% CI: 0.82–1.05). Results of this updated meta-analysis suggest that fish consumption is unlikely to be substantially associated with risk of pancreatic cancer.

## Introduction

Globally, pancreatic cancer represents the fourth leading cause of cancer death, imposing serious threats on public health [1]. Pancreatic cancer has a poor prognosis and high fatality rates, with a 5-year survival rate of less than 5%. Efficient screening methods or treatment modalities for this malignancy are still lacking, highlighting the importance of effective prevention strategies [1]. Known risk factors for pancreatic cancer include chronic pancreatitis, cigarette smoking, family history, excess body fat and diabetes [1]. Dietary factors may also pay a role in the development of pancreatic cancer, but few certainties have been achieved [2].

Both *in vitro* and *in vivo* studies have demonstrated an important role of inflammation in the initiation and progression of pancreatic cancer [3]. Multiple lines of evidence has shown that marine-derived n-3 polyunsaturated fatty acids (PUFA) such as eicosapentaenoic acid and docosahexaenoic acid have inhibition effects on a number of aspects of inflammation [4]. Therefore, it is biologically plausible that consumption of fish, a major dietary source of marine n-3 PUFA, may provide protections against pancreatic cancer. A 2012 meta-analysis [5] synthesizing results from 10 case-control studies and eight prospective cohort studies concluded that fish consumption was not associated with risk of pancreatic cancer. However, the studies included in the meta-analysis differed substantially by their methodological and population characteristics such as the statistical adjustments made and the levels of fish consumption, and how these variations may affect the evaluated association of fish and pancreatic cancer need to be addressed (e.g. in regression and dose-response analyses). Furthermore, several additional prospective studies [6–10] of fish consumption and pancreatic cancer which almost have doubled the number of cases included in the previous meta-analysis have emerged. Therefore, we carried out an update meta-analysis to comprehensively evaluate the relationship between long-term fish consumption and risk of pancreatic cancer.

## Materials and methods

### Literature search and selection

We planned, conducted, and reported this meta-analysis in concordance with the guidelines of the ‘Meta-analysis Of Observational Studies in Epidemiology group’ [11]. We performed a systematic search of literature indexed by PubMed and EMBASE databases for potentially relevant studies published up to March 30, 2019. A detailed literature search strategy is reported in S1 Table, the core search terms involved dietary factors (including fish consumption), pancreatic cancer, and study design. There were no language restrictions imposed. To identify any additional studies, we further hand screened the references of the retrieved full publications and those of the previous meta-analysis [5].

Potentially eligible studies were prospective studies (e.g. prospective cohort studies, case-cohort studies, or nested case-control studies for which diets were recorded prior to cancer diagnosis) in which the relationship between fish consumption and risk of incident or death from pancreatic cancer were examined. To be included in the meta-analysis, studies had to report risk estimates such as hazard ratio, relative risks (RR), or odds ratio with corresponding 95% confidence intervals (CI) of pancreatic cancer associated with fish consumption.

### Data extraction and quality assessment

We used a standardized data-collection form to extract the following information from each included study: the first author’s last name, year of publication, country where the study was performed, name of study (if available), length of follow-up, age and sex of participants, number of cases and participants, different categories of fish consumption and the corresponding fully adjusted risk estimates with 95% CI, and potential confounders included in the multivariate models. We used the 9-star Newcastle-Ottawa Scale (NOS) [12] to evaluate quality of the included studies, on the basis of following three major characteristics of included studies: selection of the study groups (0–4 stars), adjustment for known confounding factors (0–2 stars), and ascertainment of the outcome of interest (0–3 stars). A higher score represents better methodological quality.

### Statistical analysis

We calculated both summary risk estimates of pancreatic cancer for the highest compared with the lowest categories and for each 50-g/day increment (~0.5 servings/day [13]) of fish consumption. We combined study-specific risk estimates using a random-effects model which accounts for both within- and between-study variation [14]. For studies that reported sex-specific results instead of overall results for the whole study population, we pooled the sex-specific estimates using a fix-effect model and included the combined estimate in the meta-analysis. For one Swedish study [15] in which both results based on a single measurement of fish consumption (baseline) and those based on long-term average consumption were reported, the later estimates less prone to measurement errors were used in this meta-analysis. For one Japanese study [7] that provided both results for the whole population and those after omitting pancreatic cancer diagnosed during the first three years of follow-up, the later estimates were included in the meta-analysis because these were less likely to be affected by reverse causation.

To estimate dose-response estimate for each study, we used the method of generalized least squares trend estimation as proposed by Greenland and Longnecker [16] and Orsini *et al*.[17], which has been widely used in previous meta-analyses of nutritional studies [18–20]. For each category of fish consumption, we extracted from each study the amount of fish consumption, distributions of cases and person-years, and risk estimates of pancreatic cancer with corresponding 95% CI. When fish consumption was expressed in frequency (e.g. servings/day), we converted the consumption into weight by using 100 g as a standard portion size [13]. When the number of cases or person-years in each intake category was not reported, we estimated the data from total number of cases or person-years. The median or mean fish consumption in each category was used as the average intake amount, and when these values were not reported, the midpoint of the upper and lower boundaries was used. If the highest or lowest category was open-ended, we assumed that the width of the interval was the same as in the closest category.

We performed various stratified analyses according to following study and population characteristics: geographic location, sex, length of follow-up, number of pancreatic cancer cases, NOS quality score, and statistical adjustment for potential confounders. We performed a sensitivity analysis by omitting studies that used pancreatic cancer death as the study outcome. To examined potential nonlinearity for the association between fish consumption and risk of pancreatic cancer, we used restricted cubic splines with three knots at percentiles 10%, 50% and 90% of the distribution [21]. A *P* value for nonlinearity was calculated by testing the null hypothesis that the coefficient of the second spline is equal to zero.

Heterogeneity among studies was assessed using the *Q* and *I*^2^ statistics [22]. We set the significance level for the *Q* statistic at 0.10 instead of the more conventional level of 0.05 to avoid type II errors resulting from low statistical power. For the *I*^2^ statistic, we considered a value of <25%, 25–50% and >50% as little or no, moderate, and considerable heterogeneity, respectively. We assessed potential publication bias using both Begg rank correlation test and Egger linear regression test [23, 24]. All statistical analyses were carried out using STATA versions 12.0 and 15.1 (STATA Corp., College Station, TX, USA). All *P* values were two-sided, and the level of significance was at <0.05, unless explicitly stated.

## Results

### Study characteristics

**Fig 1:**
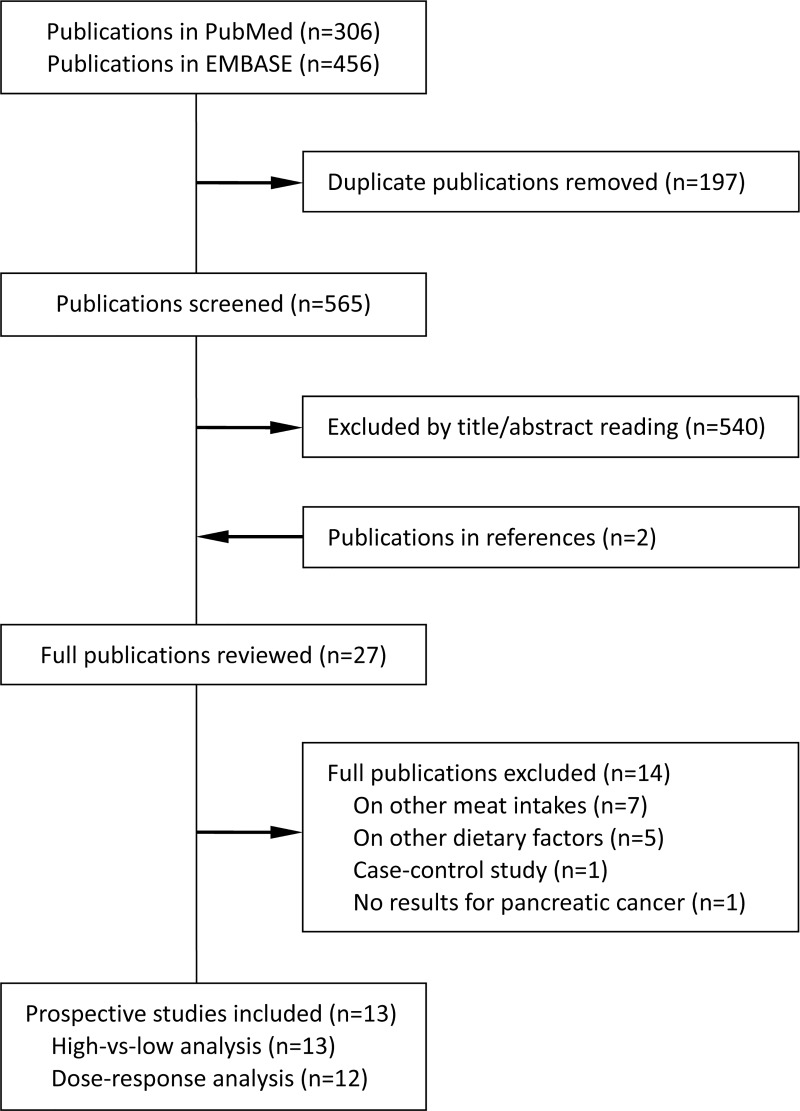
Flow chart of literature search for the mete-analysis.

Fig 1 presents a flow chart of literature screening and selection. Finally, our meta-analysis included 13 studies [6–10, 15, 25–31] that prospectively investigated the relationship between fish consumption and risk of pancreatic cancer (S2 Table). These 13 studies were published between 1993 and 2018, including 4994 pancreatic cancer cases and 1,794,601 participants. The median number of cases and participants was 300 (54 to 1156) and 82,024 (3980 to 510,314), respectively. The median length of follow-up was 11.3 (6.8 to 17.4) years. Five studies were from the US, four studies were from Asia (two from Japan and one each from China and Iran), and four studies were from Europe (one each from Finland, Sweden and the Netherland in addition to one study conducted in 10 European countries). Participants were male (n = 2) or female (n = 2) only in four studies and the remainder included both sexes. While all studies included in the meta-analysis made multivariate adjustments, the potential confounders considered in the analyses varied. Except for age (all studies) and sex (where appropriate), other commonly considered confounding factors included smoking (n = 10), alcohol consumption (n = 9), history of diabetes (n = 9), body size measurement (e.g. body mass index [BMI]; n = 8), physical activity (n = 6), and dietary energy intake (n = 11). Fewer studies further adjusted for family history of pancreatic cancer (n = 3) or consumption of red and processed meat (n = 4). Six of the 13 studies had eight or nine NOS stars, four had seven and three had fewer stars (S3 Table).

### Meta-analysis

**Fig 2:**
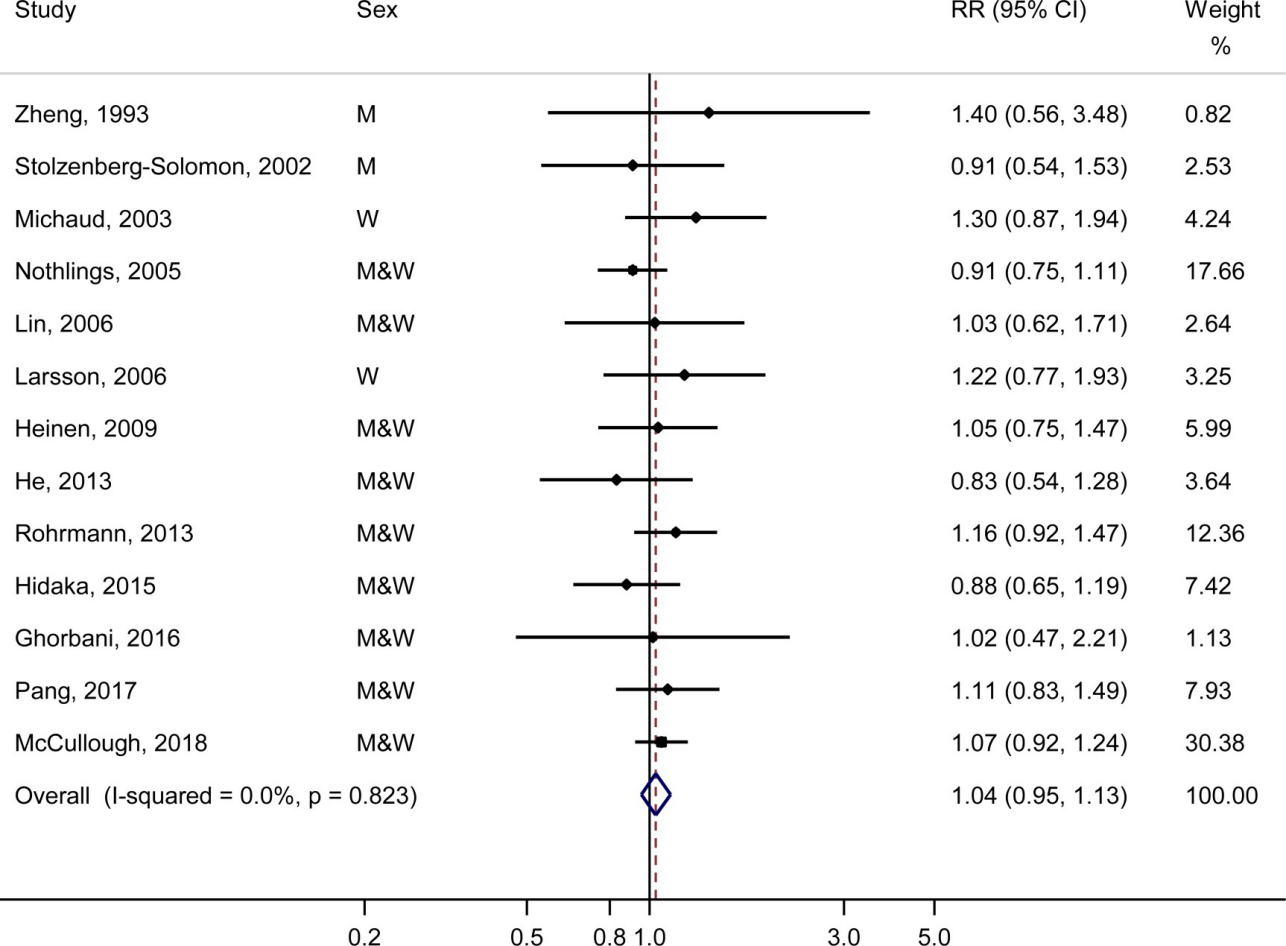
Meta-analysis of fish consumption (highest-vs-lowest) and risk of pancreatic cancer.

All studies were included in the meta-analysis of the highest vs. the lowest categories of fish consumption. The summary RR was 1.04 (95% CI: 0.95–1.13), with no evidence for heterogeneity (*P* = 0.82, *I*^2^ = 0.0%) (Fig 2). There was no evidence for publication bias (*P*_Egger_ = 0.77 and *P*_Begg_ = 1.00).

**Fig 3:**
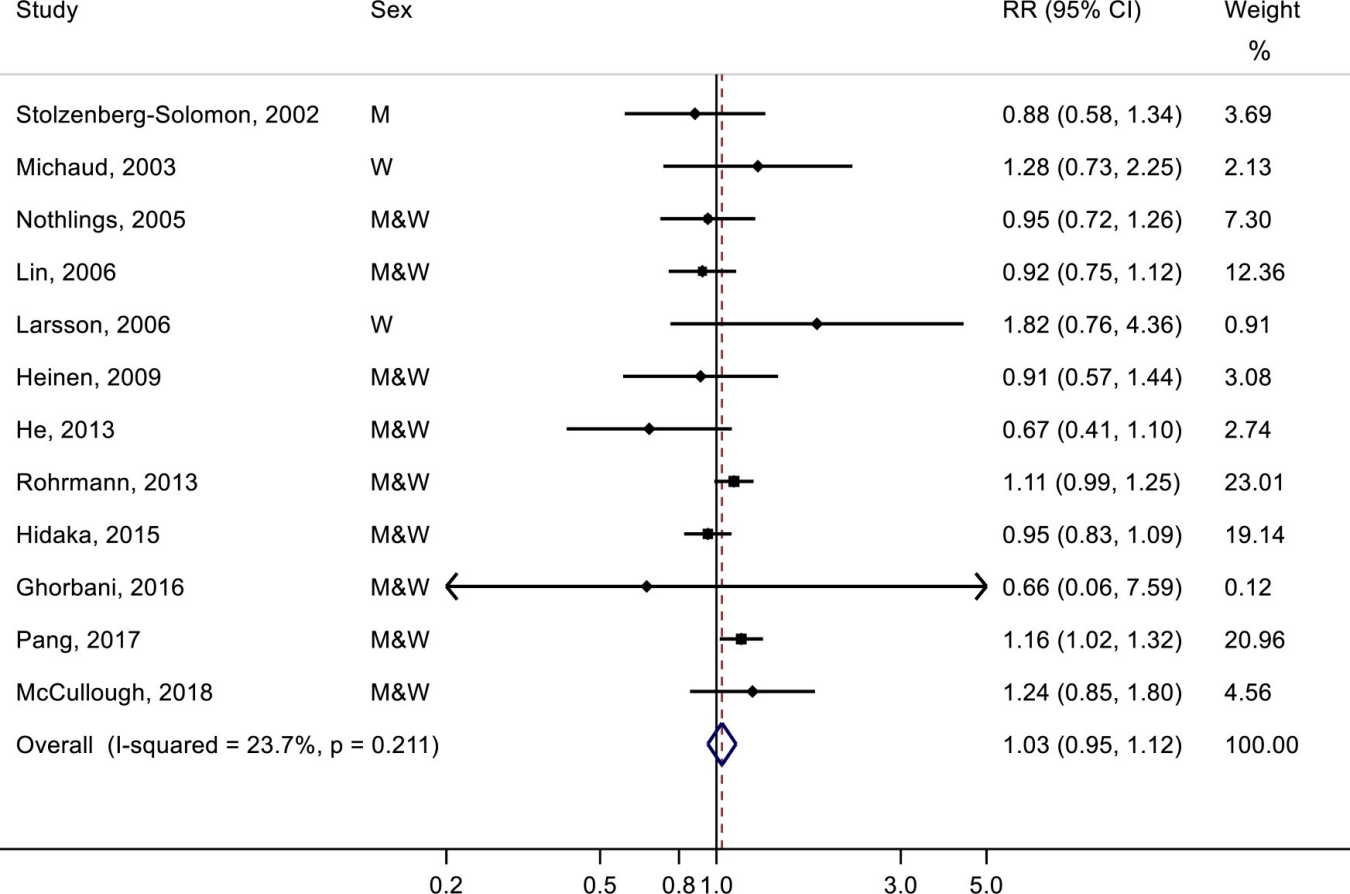
Meta-analysis of fish consumption (per 50-g/day) and risk of pancreatic cancer.

**Fig 4:**
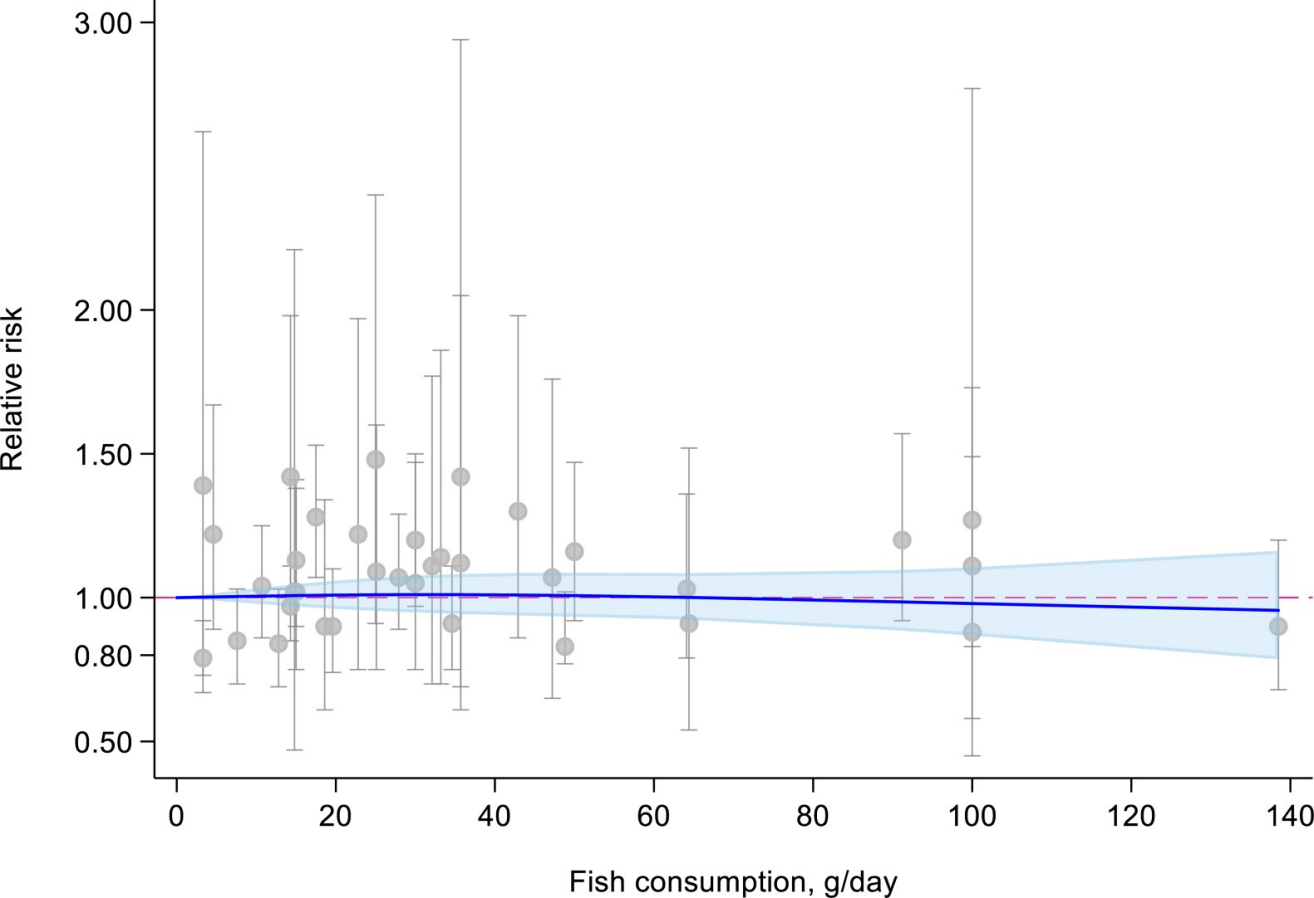
Dose-response meta-analysis of fish consumption and risk of pancreatic cancer assessed by restricted cubic splines.

One study [25] was not eligible for the dose-response analysis because the amount of fish consumption for each quartile was not available. The dose-response meta-analysis of the remaining 12 studies showed that the summary RR of pancreatic cancer for each 50-g/day increment in fish consumption was 1.03 (95% CI: 0.95–1.12), with low evidence for heterogeneity (*P* = 0.21, *I*^2^ = 23.7%) (Fig 3). There was no evidence for publication bias (*P*_Egger_ = 0.53 and *P*_Begg_ = 0.95). We found no evidence for a nonlinear relationship between fish consumption and risk of pancreatic cancer (*P*_nonlinearity_ = 0.61) (Fig 4).

### Subgroup and sensitivity analyses

We performed subgroup analyses according to a number of predefined factors, and these analyses were conducted both for the highest vs. the lowest categories and for each 50-g/day increment of fish consumption (Table 1). In general, results of the subgroup analyses did not support a significant association between fish consumption and risk of pancreatic cancer. There was a trend towards increased risk of pancreatic cancer in men in the highest-vs-lowest analysis (RR = 1.24, 95% CI: 0.99–1.54), but this result attenuated in the dose-response analysis (RR = 1.11, 95% CI: 0.97–1.27). There was a suggestion that the association between fish consumption and risk of pancreatic cancer was modified by adjustment for family history of pancreatic cancer (*P*_difference_ = 0.04 in the highest-vs-lowest analysis and 0.05 in the dose-response analysis). Fish consumption was associated with increased risk of pancreatic cancer in studies without adjustment for participants’ family history of pancreatic cancer (RR _highest-vs-lowest_ = 1.10, 95% CI: 1.00–1.21; RR_50-g/day increment_ = 1.09, 95% CI: 1.02–1.18). Conversely, there was a non-significant inverse association among studies with the adjustment (RR _highest-vs-lowest_ = 0.89, 95% CI: 0.76–1.04; RR_50-g/day increment_ = 0.93, 95% CI: 0.82–1.05) (Table 1).

**Table 1:** Subgroup analysis for the meta-analysis of fish consumption and risk of pancreatic cancer.

		Highest vs. lowest					Per 50 g/day increment				
		*N*	RR (95% CI)	P*	*I*^2^ (%)	*P*†	*N*	RR (95% CI)	P*	*I*^2^ (%)	*P*†
Geographic area											
Asia		4	1.00 (0.83–1.21)	0.76	0.0	Ref.	4	1.02 (0.88–1.17)	0.12	49.0	Ref.
Europe		4	1.11 (0.94–1.31)	0.80	0.0	0.41	4	1.09 (0.98–1.21)	0.39	0.2	0.69
USA		5	1.02 (0.90–1.15)	0.35	10.2	0.87	4	1.00 (0.78–1.29)	0.20	36.1	0.94
Sex of participants											
Men		5	1.24 (0.99–1.54)	0.30	17.6	0.51	4	1.11 (0.97–1.27)	0.25	8.7	0.84
Women		5	1.12 (0.92–1.36)	0.47	0.0		5	1.07 (0.90–1.27)	0.31	16.9	
Average duration of follow-up											
<10 years		4	0.95 (0.82–1.11)	0.64	0.0	0.21	4	0.99 (0.78–1.25)	0.13	47.6	0.92
≥10 years		9	1.08 (0.97–1.19)	0.86	0.0		8	1.03 (0.94–1.12)	0.33	13.0	
No. of cases											
<200		6	1.08 (0.87–1.32)	0.66	0.0	0.71	5	0.97 (0.70–1.34)	0.25	25.2	0.52
≥200		7	1.03 (0.94–1.13)	0.67	0.0		7	1.04 (0.96–1.14)	0.21	28.4	
NOS quality score											
<8		7	0.99 (0.87–1.13)	0.62	0.0	0.42	6	0.99 (0.85–1.15)	0.12	43.1	0.59
≥8		6	1.07 (0.96–1.19)	0.79	0.0		6	1.05 (0.95–1.16)	0.35	10.9	
Statistical adjustment											
Smoking (1)‡	*Yes*	10	1.08 (0.97–1.19)	0.91	0.0	0.21	9	1.03 (0.95–1.11)	0.42	2.1	0.89
	*No*	3	0.95 (0.82–1.11)	0.44	0.0		3	0.98 (0.75–1.28)	0.06	64.0	
Smoking (2)§	*Yes*	7	1.07 (0.96–1.18)	0.76	0.0	0.39	7	1.04 (0.96–1.13)	0.52	0.0	0.83
	*No*	6	0.99 (0.86–1.13)	0.65	0.0		5	1.00 (0.83–1.21)	0.06	56.4	
Alcohol	*Yes*	9	1.01 (0.92–1.11)	0.77	0.0	0.27	8	1.03 (0.90–1.17)	0.15	34.5	0.99
	*No*	4	1.14 (0.95–1.36)	0.73	0.0		4	1.04 (0.92–1.18)	0.30	18.8	
Physical activity	*Yes*	6	1.06 (0.93–1.22)	0.50	0.0	0.69	6	1.05 (0.94–1.18)	0.12	42.2	0.42
	*No*	7	1.02 (0.92–1.13)	0.81	0.0		6	0.97 (0.85–1.11)	0.52	0	
Body size	*Yes*	8	1.07 (0.97–1.18)	0.70	0.0	0.25	8	1.08 (0.97–1.20)	0.16	34.0	0.12
	*No*	5	0.96 (0.82–1.11)	0.86	0.0		4	0.92 (0.80–1.07)	0.99	0.0	
Diabetes	*Yes*	9	1.03 (0.95–1.13)	0.61	0.0	0.73	9	1.05 (0.96–1.15)	0.24	22.9	0.36
	*No*	4	1.09 (0.83–1.42)	0.79	0.0		3	0.95 (0.76–1.18)	0.31	14.4	
Family history of PC	*Yes*	3	0.89 (0.76–1.04)	0.93	0.0	0.04	3	0.93 (0.82–1.05)	0.40	0.0	0.049
	*No*	10	1.10 (1.00–1.21)	0.99	0.0		9	1.09 (1.02–1.18)	0.48	0.0	
Dietary energy	*Yes*	11	1.03 (0.94–1.12)	0.70	0.0	0.69	10	1.02 (0.93–1.12)	0.33	11.8	0.69
	*No*	2	1.09 (0.85–1.40)	0.80	0.0		2	1.05 (0.84–1.31)	0.06	72.5	
Red/processed meat	*Yes*	4	0.99 (0.83–1.18)	0.46	0.0	0.60	4	1.02 (0.82–1.26)	0.03	66.5	0.96
	*No*	9	1.05 (0.96–1.15)	0.80	0.0		8	1.05 (0.96–1.14)	0.61	0.0	

Abbreviations: CI, confidence interval; N, number of studies; NOS, Newcastle Ottawa Scale; PC, pancreatic cancer; RR, relative risk.

*P for heterogeneity.

†*P* for difference.

‡Any adjustment for smoking.

§Amount/duration of smoking was included as a continuous variable or had ≥3 categories.

There were two studies [25, 29] that used pancreatic cancer death at the study outcome. However, omitting both studies yielded results that were similar to those from the overall meta-analyses (RR _highest-vs-lowest_ = 1.03, 95% CI: 0.95–1.13; RR_50-g/day increment_ = 1.05, 95% CI: 0.96–1.15).

## Discussion

In this updated meta-analysis of 13 prospective studies including approximately 1.8 million participants and 5000 pancreatic cancer cases, long-term fish consumption was not significantly associated with risk of pancreatic cancer. Our findings were concordance with results from a previous meta-analysis that included eight prospective studies with only 1853 pancreatic cancer cases and 0.54 million participants [5]. Our meta-analysis updated the evidence by adding five prospective studies with 3141 cases and 1.26 million participants and, therefore, had sufficient statistical power to detect any moderate association between fish consumption and risk of pancreatic cancer.

In this meta-analysis, we conducted both the highest-vs-lowest and the dose-response analyses, and did not find any evidence of a nonlinear relationship between fish consumption and risk of pancreatic cancer. Furthermore, we were able to address potential influences of important study characteristics (e.g. statistical adjustments for important confounders) on the examined association by performing various stratified and regression analyses. Overall, results of these analyses confirmed that fish consumption was not substantially associated with risk of pancreatic cancer. The only exception is that we found increased risk of pancreatic cancer associated with fish consumption in studies that were not adjusted for family history of pancreatic cancer and a tendency towards decreased risk in studies with such an adjustment. While these stratified results may have occurred by change due to the multiple analyses performed, they may also result from reverse causation. Family history of pancreatic cancer is a strong risk factor for pancreatic cancer and a familial basis has been observed in approximately 10% of pancreatic cancer cases [1]. As a result, participants having a familial basis may have changed their dietary habits, for example increasing consumption of fish and other foods thought to be anti-carcinogenic, which may attenuate or even reverse any inverse association between fish consumption and risk of pancreatic cancer. Therefore, a modest inverse relationship between fish consumption and risk of pancreatic cancer remains possible and future studies with careful adjustments for potential confounders including family history of pancreatic cancer are needed.

Strengths of this meta-analysis include the prospective nature of the included studies, the large number of cases involved, and the comprehensive statistical analyses that we performed. The limited heterogeneity observed across most of our analyses and the concordance between the summary results and results from most of the primary studies further exclude a substantial association between fish consumption and risk of pancreatic cancer.

One limitation of this meta-analysis and most original studies included is that potential influences of preparation methods of fish on risk of pancreatic cancer have not been evaluated. Recent results from large US prospective studies suggested that meat prepared with different methods may have different impacts on risk of type 2 diabetes [32], a potential risk factor for pancreatic cancer [1]. It is possible that certain carcinogenic substances produced by the high-temperature cooking process may also diminish any benefits of fish on the prevention of pancreatic cancer [33]. For example, in a US prospective study [31], consumption of non-fried fish, but not shellfish or fried fish, was associated with substantially lower risk of pancreatic cancer. Another common limitation in meta-analyses and cohort studies of diets and disease risk is that dietary information was mostly collected using food frequency questionnaires and only a single collection was done at baseline. Therefore, misclassification of diets is inevitable, which likely to be non-differential and attenuate the examined relationship between fish consumption and pancreatic cancer risk. Last but not least, this meta-analysis of observational studies is subject to residual confounding as was any original studies included. However, residual confounding is likely to strengthen rather than mask the examined association because fish is widely considered a part of a healthy diet (e.g. the Mediterranean diet).

Of note is that our overall null findings do not exclude the possibility that intake or fish or omega-3 fatty acids may be associated with a specific subtype of pancreatic cancer. Different histological or molecular subtypes of pancreatic cancer may differ substantially in terms of their initiation, evolution, clinical features, and responses to treatments [34, 35]. It is thus plausible that the etiological role of environmental factors (including diet) on the development of pancreatic cancer may be tumor-subtype-specific. In this case, the emerging molecular pathological epidemiology (MPE) may shed some novel light on the etiological role of exogenous and endogenous environments in the development and progress of heterogeneous cancers [36, 37]. MPE is a relatively new transdisciplinary field that can link environmental factors (e.g. diet, lifestyle, and host microbiome) to molecular pathology of cancer, and it has been applied to studies of many cancers including breast, lung, and colorectal, pancreatic cancers [36, 38, 39]. In the context of the remarkable technical advances in multi-omics (e.g. genomics, metabolomics, proteomics, immunomics, and microbiome), MPE can further contribute to precise medicine and public health by improving our understanding of disease-associated pathological processes and molecular pathways, which subsequently facilitates personalized prevention, early detection, and management of cancer [36].

In summary, results of this large updated meta-analysis suggest that fish consumption is not significantly associated with risk of pancreatic cancer. Accumulative evidence has suggested that red and processed meat may increase risk of various digestive malignancies, in particular with colorectal, esophageal and gastric cancers [40]. A recent meta-analysis of cohort studies also suggested that consumption of red and processed meat was associated with increased risk of pancreatic cancer in men [41]. Despite that our and other studies [42] showed no or only weak-to-moderate inverse associations between fish consumption and risk of the aforementioned cancers, dietary fish is still a good source of animal protein and can be recommended as a substitution for red and processed meat in regular diets for cancer prevention.

## Supporting information

S1 Checklist PRISMA checklist. (DOCX)

S1 Table Literature search strategies in the databases. (DOCX)

S2 Table Characteristics of the included prospective studies on fish consumption and risk of pancreatic cancer. (DOCX)

S3 Table The quality of included studies assessed by the Newcastle Ottawa Scale. (DOCX)
