# Supplementary material for: Meta-analysis of fish consumption and risk of pancreatic cancer in 13 prospective studies with 1.8 million participants
Source: PLoS One. 2019 Sep 6;14(9):e0222139. doi: 10.1371/journal.pone.0222139 (PMC6730991; doi:10.1371/journal.pone.0222139)
Supplement: S1 Table — (DOCX) [file pone.0222139.s002.docx]

| **PubMed** |
| --- |
| (diet*[tiab] OR food*[tiab] OR meat OR fish OR seafood OR shellfish) AND (pancreatic cancer OR pancreas cancer OR pancreatic carcinoma) AND (cohort OR prospective OR follow-up study) NOT ((retrospective studies[mh]) OR (review[pt])) |
| **EMBASE** |
| ('diet'/exp OR 'dietary intake'/exp OR 'dietary habit'/exp OR 'food'/exp OR meat OR fish OR seafood OR shellfish) AND ('pancreatic cancer' OR 'cancer of pancreas' OR 'pancreatic carcinoma') AND (cohort OR prospective OR 'follow up') NOT (('retrospective study'/exp) OR ('review'/exp)) |

**S1 Table:** Literature search strategies in the databases
