# Supplementary material for: Meta-analysis of fish consumption and risk of pancreatic cancer in 13 prospective studies with 1.8 million participants
Source: PLoS One. 2019 Sep 6;14(9):e0222139. doi: 10.1371/journal.pone.0222139 (PMC6730991; doi:10.1371/journal.pone.0222139)
Supplement: S2 Table — (DOCX) [file pone.0222139.s003.docx]

**S2 Table.** Characteristics of the included prospective studies on fish consumption and risk of pancreatic cancer

| **Author, year (country)** | **Study population (duration)*** | **Participants** | **No. of cases** | **Comparison, highest vs. lowest** | **RR (95%CI)** | **Statistical adjustment** |
| --- | --- | --- | --- | --- | --- | --- |
| Zheng, 1993†  (USA) | Life-insurance policy holders (16.3 yr) | 17,633 M aged ≥35 yr | 57 PC deaths | 4^th^ vs. 1^st^ quartile | 1.4 (0.6-3.7) | Age, smoking (never, current <25 cigarettes/d or ex-smokers, or current ≥25 cigarettes/d), and intake of alcohol and dietary energy intake. |
| Stolzenberg-Solomon, 2002  (Finland) | ATBC (10.2 yr) | 27,111 male smokers aged 50-69 yr | 163 incident PC | >55.8 vs. 0 g/d | 0.91 (0.54-1.52) | Age, years of smoking, and dietary energy intake. |
| Michaud, 2003  (USA) | NHS (17.4 yr) | 88,802 female nurses aged 30-55 yr | 178 incident PC | ≥2 serv/wk vs. <4 serv/mo | 1.30 (0.86-1.98) | Age, pack-years of smoking (past 15 years; current and former smokers separately), physical activity, BMI, height, diabetes, menopausal status, and dietary energy intake. |
| Nöthlings, 2005 (USA) | MCS (7.4 yr) | 190,545 M&W aged 45-75 yr | 482 incident PC | 17.4 vs. 1.1 g per 2000 kcal/d§ | 0.91 (0.75-1.11) | Age, ethnicity, diabetes, family history of PC, smoking (never, former, or current), and dietary energy intake. |
| Lin, 2006  (Japan) | JACC ( 10.0 yr) | 105,438 M&W aged 40-79 yr | 300 PC deaths | Almost daily vs. 0-2 serv/mo | 1.27 (0.58-2.77)M  0.88 (0.45-1.73)W | Age, study area, and smoking (nonsmokers or smokers [1-19 or ≥20 pack-years]). |
| Larsson,2006  (Sweden) | SMC (15.3 yr) | 36616 W | 172 incident PC | ≥2.0 vs. ≤1.0/wk | 1.22 (0.77-1.92) | Age, education, BMI, smoking (never, former[<20 or ≥20 pack-years, current [<20 or ≥20 pack-years], or missing), and intakes of alcohol, folate, red meat, processed meat, poultry, egg and dietary energy. |
| Heinen, 2009  (The Netherlands) | NLCS sub-cohort (13.3 yr) | 3980 M&W aged 55-69 yr | 350 incident PC | 32.3(M) or 28.2(W) vs. 0 g/d‡  Per 120 g/wk | 1.05 (0.75-1.47)  0.97 (0.81-1.11) | Age, sex, smoking (current or not, number of cigarettes/d, and number of years of smoking), diabetes, hypertension, and intake of alcohol, vegetables, fruit and dietary energy. |
| He, 2013  (USA) | VITAL (6.8 yr) | 66,616 M&W aged 50-76 yr | 151 incident PC | ≥2.25 vs. <1.23 serv/wk  Per 1 serv/wk | 0.83 (0.54-1.28)  0.89 (0.77-1.02) | Age, sex, ethnicity, education, BMI, physical activity, smoking (never, former [stopped >10 years or ≤10 years], or current), diabetes, family history of PC, NSAID use, and intake of alcohol, fruit, vegetables, dairy, red/processed meat and dietary energy; further adjusting for fried fish and shellfish (for non-fried fish) or fried fish and non-fried fish (for shellfish), or non-fried fish and shellfish (for fried fish). |
| Rohrmann, 2013  (10 European countries) | EPIC ( 11.3 yr) | 477,202 M&W aged 35-75 yr | 865 incident PC | ≥ 40 vs. <10 g/d  Per 50 g/d | 1.16 (0.92-1.47)  1.11 (0.99-1.25) | Age, sex, study area, height, weight, physical activity, smoking (never, former stopped <10 years, former stopped ≥10 years, current 1-14, 15-24, or >25 cigarettes/d, other, or missing), education, diabetes, and dietary energy intake. |
| Hidaka, 2015  (Japan) | JPHC (13.0 yr) | 82,024 M&W aged 45-74 yr | 378 incident PC | 138.5 vs. 36.8 g/d‡ | 0.90 (0.68-1.20) | Age, sex, study area, BMI, smoking (never, former, current [<20, 20-40 or ≥40 cigarettes/d], or missing), physical activity, diabetes, family history of PC, and intake of alcohol, red /processed meat and dietary energy. |
| Ghorbani, 2016  (Iran) | GCS (7.7 yr) | 50,054 M&W aged ≥40 yr | 54 incident PC | 14.8 vs. 0 g/d‡ | 1.02 (0.47-2.21) | Age, sex, study area, BMI, education, diabetes, pack-years of smoking, opium use, physical activity, wealth score, and intake of alcohol and dietary energy. |
| Pang, 2017  (China) | CKB (8.1 yr) | 510,314 M&W aged 30-79 yr | 688 incident PC | Daily vs. never/rarely | 1.11 (0.83-1.49)M&W  1.69 (1.17-2.43)M  0.77 (0.47-1.27)W | Age, sex (not for sex-specific analyses), study area, education, smoking (never, occasional, ever regular), alcohol, BMI, physical activity, diabetes (not for sex-specific analyses), and intake of other foods (fresh fruit, soybean products, red meat, poultry, dairy, and preserved vegetables; not for sex-specific analyses). |
| McCullough, 2018  (USA) | CPS-II (15.7 yr) | 138,266 M&W | 1156 incident PC (81.4% were PC death) | >1.6-2.6 vs. ≤0.6 serv/wk in M; >1.4-2.2 vs. ≤0.5 serv/wk in W | 1.07 (0.89-1.20)M&W  1.07 (0.83-1.38)M  1.08 (0.83-1.42)W | Age, sex (not for sex-specific analyses), BMI, diabetes, smoking (never, former [≤ 5, > 5-10,> 10-15, or > 15 years since quit], or current [< 20, ≥ 20 cigarettes/d, or missing]), and intake of alcohol, saturated fat and dietary energy. |

ATBC, Alpha-Tocopherol, Beta-Carotene (Cancer Prevention Study); BMI, body mass index; CKB, China Kadoorie Biobank; CPS, Cancer Prevention Study; EPIC, European Prospective Investigation into Cancer (and Nutrition Study); GCS, Golestan Cohort Study; JACC, Japan Collaborative Cohort; JPHC, Japan Public Health Cente; NHS, Nurses’ Health Study; NLCS, the Netherlands Cohort Study; NSAID, nonsteroidal anti-inflammatory drug; M, men; MCS, Multiethnic Cohort Study; mo, month; serv, servings; SMC, Swedish Mammography Cohort; PC, pancreatic cancer; VITAL, VITamins And Lifestyle; wk, week; yr, years;

*Mean years of follow-up, which were either reported in the articles or calculated by dividing the number participants by the total number of person-years.

†Only included in the high-vs-low analysis because there were insufficient data to calculate the dose-response estimates.

‡Median intake values.
