# Supplementary material for: Meta-analysis of fish consumption and risk of pancreatic cancer in 13 prospective studies with 1.8 million participants
Source: PLoS One. 2019 Sep 6;14(9):e0222139. doi: 10.1371/journal.pone.0222139 (PMC6730991; doi:10.1371/journal.pone.0222139)
Supplement: S3 Table — (DOCX) [file pone.0222139.s004.docx]

**S3 Table.** The quality of included studies assessed by the Newcastle Ottawa Scale

| Study | **Selection** | | | | **Comparability** | **Outcome** | | | **Total stars** |
| --- | --- | --- | --- | --- | --- | --- | --- | --- | --- |
|  | Representativeness  of exposed cohort | Selection of the non- exposed  cohort | Ascertainment of exposure | Demonstration that outcome of interest was not present at start of study | Comparability  of cohorts on the basis of the design or analysis | Assessment of outcome | Was follow-up long enough for  outcomes to  occur | Adequacy of follow up of cohorts |  |
| Zheng, 1993 | 0 | 1 | 1 | 0 | 2 | 0 | 1 | 0 | **5** |
| Stolzenberg-Solomon, 2002 | 0 | 1 | 1 | 1 | 2 | 1 | 1 | 0 | **7** |
| Michaud, 2003 | 0 | 1 | 1 | 1 | 2 | 1 | 1 | 0 | **7** |
| Nöthlings, 2005 | 1 | 1 | 1 | 1 | 2 | 1 | 0 | 0 | **7** |
| Lin, 2006 | 1 | 1 | 0 | 1 | 1 | 0 | 1 | 1 | **6** |
| Larsson,2006 | 1 | 1 | 1 | 1 | 2 | 1 | 1 | 0 | **8** |
| Heinen, 2009 | 1 | 1 | 1 | 1 | 2 | 1 | 1 | 1 | **9** |
| He, 2013 | 1 | 1 | 1 | 1 | 1 | 1 | 0 | 1 | **7** |
| Rohrmann, 2013 | 1 | 1 | 1 | 1 | 2 | 1 | 1 | 1 | **9** |
| Hidaka, 2015 | 1 | 1 | 1 | 1 | 2 | 1 | 1 | 1 | **9** |
| Ghorbani, 2016 | 1 | 1 | 1 | 1 | 2 | 1 | 1 | 0 | **8** |
| Pang, 2017 | 1 | 1 | 0 | 1 | 1 | 1 | 0 | 1 | **6** |
| McCullough, 2018 | 1 | 1 | 1 | 1 | 2 | 1 | 1 | 1 | **9** |
|  | | | | | | | | | |
